# Producing valid statistics when legislation, culture and medical practices differ for births at or before the threshold of survival: report of a European workshop

**DOI:** 10.1111/1471-0528.15971

**Published:** 2019-11-06

**Authors:** LK Smith, B Blondel, J Zeitlin, on behalf of the Euro‐Peristat Scientific Committee, Gerald Haidinger, Sophie Alexander, Urelija Rodin, Theopisti Kyprianou, Petr Velebil, Laust Mortensen, Luule Sakkeus, Mika Gissler, Günther Heller, Nicholas Lack, Aris Antsaklis, István Berbik, Helga Sól Ólafsdóttir, Sheelagh Bonham, Marina Cuttini, Janis Misins, Jelena Isakova, Yolande Wagener, Miriam Gatt, Jan Nijhuis, Katarzyna Szamotulska, Henrique Barros, Mihai Horga, Jan Cap, Natasa Tul, Francisco Bolúmar, Karin Gottvall, Karin Källén, Sylvan Berrut, Mélanie Riggenbach, Alison Macfarlane, Marie Delnord, Mélanie Durox, Ashna Hindori‐Mohangoo

**Affiliations:** ^1^ Department of Health Sciences College of Life Sciences University of Leicester Leicester UK; ^2^ Inserm UMR 1153 Obstetrical, Perinatal and Pediatric Epidemiology Research Team (Epopé) Centre for Epidemiology and Statistics Sorbonne Paris Cité DHU Risks in pregnancy Paris Descartes University Paris France

Perinatal mortality is a major population health indicator conveying important signals about the state of maternity care and measures of the current and future health of mothers and newborns. International comparisons are used to encourage countries to improve their perinatal health and health systems. However, extensive evidence highlights methodological challenges to ensuring valid and robust comparisons, as a lack of standardised criteria can lead to bias and inappropriate inferences.^1^ One major issue is the wide international variation in the criteria for classification and registration of deaths as a stillbirth or neonatal death at the threshold of survival.^2^, ^3^, ^4^, ^5^ Standard practice is to minimise this problem by using a gestational age cut‐off of 24 or even 28 weeks for mortality rate calculations. However, this strategy excludes a significant number of stillbirths, at least one in five deaths before 24 weeks of gestation and over one in three deaths before 28 weeks.^6^ As the gestational age limit for initiation of neonatal care decreases,^7^ exclusion of these stillbirths limits the full evaluation of care provision and outcomes at early gestational ages. Further, it underestimates the burden of loss on parents’ mental and physical health.^8^, ^9^

To identify ways to improve the comparability of data on early gestational age births, a workshop was held in Kerkrade, the Netherlands (April 2018), by the Euro‐Peristat network.^10^ This European collaboration of 31 countries was set up to monitor perinatal health internationally by developing a list of valid and reliable indicators. Workshop participants comprised statisticians from national birth and death registers, obstetricians, midwives, neonatologists, epidemiologists, and population health researchers (Appendix S1). Discussion in small groups about national practices was structured around clinical scenarios to raise awareness about how legal requirements and clinical management affect registration and recording of deaths. Scenarios focused on antepartum death and preterm rupture of membranes, and explored the impact of multiple pregnancy, termination of pregnancy, induction of labour, and assessment of signs of life on recorded outcomes (Box S1). Results of the discussions were synthesised through a plenary presentation and participants provided comments on a written summary of the findings. This commentary summarises the workshop discussion and makes recommendations for the reporting of births at the threshold of survival in Europe (Box 1) in light of the 2015 Canadian Consensus Conference, which explored improving fetal death registration procedures.^11^

Box 1 **Key standards to reduce international variation in reporting of deaths at or before the threshold of survival to improve comparability of mortality rates.**

**Minimum standards for international mortality rate comparisons**

Reporting rates of mortality (stillbirth and neonatal death) from 22 weeks’ gestational age.

Ability to exclude terminations of pregnancy.

Ability to provide mortality rates by gestational age sub‐groups.

**Data requirements**

Recording of all births and deaths from at least 22 weeks’ gestational age in vital statistics or medical birth registers.

Recording of gestational age at birth for all births and deaths.

Identification of and ability to exclude terminations of pregnancy at ≥22 weeks.

**Aspirational standards for international mortality rate comparisons**

Reporting mortality rates based on alternative denominators: all births, births alive at onset of labour, births surviving to 1 day of life.

Use of a lower gestational age reporting threshold, at least 20 weeks.

**Data requirements**

Identification of antepartum and intrapartum fetal deaths.

Survival time of live births reported in hours.

Reporting of all pregnancy outcomes from at least 20 weeks’ gestational age.

**Key approaches to achieving standards**

Use a combination of medical registers and official birth and death registrations for complete ascertainment of births and deaths, including sources such as registrations of terminations of pregnancy and births in gynaecology units and emergency departments.

Include ICD10 cause of death and other clinical data from death certificates and medical or hospital registers to facilitate classification of deaths as intrapartum or antepartum deaths.

Recording time of death in hours for all neonatal deaths.

Lobby for consistent approach to registration and access to aid, leave, and services based on gestational age rather than signs of life.

Establishment of guidelines to increase consistency in the assessment of signs of life at or before the threshold of survival to increase internationally consistency of operationalisation of the WHO definition of live birth.

## Common international thresholds for reporting of births and deaths

Consensus recommendations to achieve a full population cohort of all live births and stillbirths from 22 weeks’ gestational age as recommended by WHO (https://icd.who.int/dev11/l-m/en#/http://id.who.int/icd/entity/914150644) as well as Euro‐Peristat were discussed. This definition was seen as achievable in Europe, as most of the 31 participating countries register fetal deaths from 22 weeks’ gestation and live births of any gestation. However, some countries still have higher gestational age thresholds for legal registration of fetal deaths (Bulgaria: 26 weeks, UK: 24 weeks, and Italy: 180 days). Some countries register fetal deaths based on birthweight criteria only or based on gestation but with a birthweight threshold of 500 g (Austria, Belgium, Czech Republic, Germany, and Poland) so births from 22 weeks’ gestation below 500 g in weight are not systematically registered. In France, registration of stillbirths is voluntary from 15 weeks. One way to fill the gaps in statutory registration data is to use data from medical registers or other sources. For instance, data on stillbirths from 22 weeks are available in Italy via a spontaneous abortion register and in the UK through national perinatal mortality surveillance. In France, Euro‐Peristat data come from administrative hospital data. These data sources make a full population cohort from 22 weeks achievable (see Smith et al.^6^ for available data used in Euro‐Peristat).

It was noted that comparability of data from 22 weeks’ gestation is reliant on the ability to exclude deaths following termination of pregnancy from reported rates or at the bare minimum to acknowledge where registrations include terminations. These deaths have a different origin to other perinatal death and their inclusion significantly changes the population cohorts and consequently the rates of stillbirth at early gestational ages, especially before 24 weeks.^12^ In most countries where late terminations are legal, fetal deaths following termination of pregnancy are registered and can be distinguished (see Blondel et al.^12^ for further detail). In some countries, however, the definition of registrable fetal deaths excludes those following termination of pregnancy.

Obtaining information on all fetal deaths from 20 weeks, as recommended in Canada,^1^ was regarded as much more challenging, but aspirational, as understanding a wider scope of pregnancy loss is important for improving reproductive outcomes. In most countries with later gestational age registration cut‐offs, a combination of registration data with medical registers would be necessary to achieve this aim. There are major challenges to achieving complete ascertainment of these deaths, particularly for those occurring outside midwifery and obstetric units such as emergency or gynaecology departments.

## Recording the timing of fetal death

Participants discussed whether it would be possible to identify the gestational age at the time of fetal death rather than the timing of the birth, as suggested by the Canadian Consensus Conference.^11^ Only the UK reported collection of information on gestation when *in utero *death was confirmed, in addition to gestation at birth for fetal deaths as part of their national perinatal mortality surveillance. For other countries, identifying gestation at confirmation of death would mean the instigation of systems to collect this information from medical notes, as it is not available through registration or current electronic medical records. Furthermore, participants expressed concerns that even in medical notes, this information could be missing or unreliable. An alternative target, which would be more achievable but still challenging for many countries, is to distinguish between intrapartum and antepartum fetal deaths. This would facilitate identification of a population cohort of live births and fetal deaths where the baby is alive at the onset of the birth process. This information is available from registration data in some countries that have introduced specific death certificates for stillbirths or perinatal deaths (including Croatia, Estonia, Latvia, Lithuania, Norway, Spain [Valencia only], UK) which may have the potential to provide information to determine whether fetal deaths occurred in the antepartum or intrapartum period. This is not routinely collected in other countries but could potentially be obtained through medical records relating to the cause of death and reasons for induction of labour associated with antepartum fetal death.

## Accounting for variation in reporting of signs of life at or before the threshold of survival

The variation in categorisation of deaths as a stillbirth or neonatal death has a major impact on estimation of both overall mortality^2^, ^3^ and gestation‐specific mortality rates.^13^ Discussion highlighted differences in the interpretation of signs of life at the threshold of survival, despite general use of WHO guidelines based on vital signs of life. These differences were considered to be closely related to local views regarding initiation of neonatal care. Some countries (Luxembourg, Netherlands) highlighted that parents’ wishes can be included in the decision whether a baby is reported as liveborn or not. Although most countries reported that guidelines existed in their country regarding initiation of neonatal intensive care for births at or before the threshold of survival, no country reported guidance that aided interpretation of the WHO definition of signs of life. In the UK, consensus guidelines are being developed regarding the assessment of signs of life to reduce national variation in practice. Such work at an international level was seen as challenging but aspirational.

Further improvements in comparisons could be facilitated in the intermediate term by collecting information on the timing of fetal deaths as antepartum and intrapartum as discussed earlier and, in addition, information on the survival time of neonatal deaths and where they occurred (labour ward or neonatal unit). This would allow identification of babies with extremely short survival times on the labour ward and could facilitate alternative reliable and robust cohort definitions such as all births alive at onset of labour or births surviving more than 1 hour. Such a definition would overcome legal registration differences but impacts such as variation in the quality of data between hospitals and additional clinician workload need to be borne in mind.

Clinicians and parents are often not aware of the overall consequences of registration of the baby as a live or stillbirth. Participants discussed the impact of legislation and other factors leading to differentials in access to maternity and paternity pay and leave, funeral costs, bereavement care, and official birth and death registration based on whether the death is reported as a stillbirth or neonatal death. For example, the requirement for a funeral differed for stillbirths and neonatal deaths, and in some countries this leads to a higher financial burden for parents in the case of neonatal death.^14^ Access to maternity and paternity pay and leave may be different based on the type of registration. For example, in the UK, parents of babies born before 24 weeks’ gestation are only eligible for maternity or paternity leave if the baby is liveborn and so a clinician’s decision to look for signs of life may be partially dependent on their awareness of this legal difference.

The participants strongly felt that the effect on parents of losing a baby should be acknowledged irrespective of whether the baby was born showing no signs of life or was born alive but died soon after. There was a call for harmonisation of practices for these early deaths, both stillbirths and neonatal deaths, relating to maternity benefits, registration, and funerals. International agreement could potentially facilitate national changes to improve care and financial provision for parents in these cases. These impacts turn a clinical issue (i.e. when did the death occur) into a social one and national lobbying to attain policies that treated stillbirth in the same way as neonatal death was seen as essential by the participants. These changes could also improve the accuracy and consistency of reporting of births by vital status.

## Conclusions

Bringing together researchers, clinicians, policy makers, and registration specialists from across Europe confirmed continuing variation in birth and death registration at or before the threshold of survival in European countries. It highlighted subtle nuances in reporting practices that are frequently overlooked and unrecognised but which may have a significant impact on comparisons of mortality rates. This type of work was seen as vital to ensure that international comparisons are robust and valid, and prevent inappropriate conclusions regarding care provision, which may have considerable financial and social implications. The working group identified minimum and aspirational standards, which we hope, will guide initiatives to improve national reporting and facilitate enhanced international monitoring and comparisons, and ultimately lead to improvements in perinatal care.

### Disclosure of interests

LS reports grants from NIHR during the conduct of the study. BB and JZ declare no competing interests. Completed disclosure of interest forms are available to view online as Supporting Information.

### Contribution to authorship

LS, BB, and JZ contributed to the overall conception and design of the workshop. LS wrote the first draft of the manuscript. LS, BB, and JZ contributed to the drafting of the manuscript, and read and approved the final manuscript. LS is the guarantor.

### Details of ethics approval

Not required.

### Funding

The Euro‐Peristat project currently receives funding from the European Commission as part of the InfAct (Information for Action) Joint Action (Consumers, Health, Agriculture and Food Executive Agency (CHAFEA) Grant no. 801553). LKS is funded by a National Institute for Health Research Career Development Fellowship. This article presents independent research funded by the National Institute for Health Research (NIHR). The views expressed are those of the authors and not necessarily those of the National Health Service, the NIHR or the Department of Health and Social Care.

### Acknowledgements

We would like to thank everyone who attended the Euro‐Peristat ‘Registration of births and deaths at the limit of viability’ workshop at Abdij Rolduc Abbey, Kerkrade, the Netherlands, for their participation and for reviewing the manuscript.

## Supporting information


**Appendix S1** **. **List of participants in the workshop on ‘Factors affecting the comparability of data sources: Birth & death registration at the limits of viability’

**Box S1** **. **Clinical scenarios used for discussion of birth and death reporting practices.

## Appendix 1

**Euro‐Peristat Scientific Committee Members:** Austria – Gerald Haidinger (The Medical University of Vienna, Vienna); Belgium – Sophie Alexander (Université Libre de Bruxelles, Brussels); Bulgaria – Rumyana Kolarova (National Centre of Public Health and Analyses, Sofia); Croatia – Urelija Rodin (Croatian National Institute of Public Health, Zagreb); Cyprus – Theopisti Kyprianou (Ministry of Health, Nicosia); Czech Republic – Petr Velebil (Institute for the Care of Mother and Child, Prague); Denmark – Laust Mortensen (University of Copenhagen, Copenhagen); Estonia – Luule Sakkeus (Tallinn University, Tallinn); Finland – Mika Gissler (National Institute for Health and Welfare, Helsinki); France – Béatrice Blondel (National Institute of Health and Medical Research [INSERM] U1153, Paris); Germany – Günther Heller (Federal Institute for Quality Assurance and Transparency in Healthcare, Berlin), Nicholas Lack (Bavarian Institute for Quality Assurance, Munich); Greece – Aris Antsaklis (University of Athens, Athens); Hungary – István Berbik (MedCongress Ltd, Budapest); Iceland – Helga Sól Ólafsdóttir (Landspitali University Hospital, Reykjavík); Ireland – Sheelagh Bonham (Healthcare Pricing Office, Dublin); Italy – Marina Cuttini (Bambino Gesù Children's Hospital, Rome); Latvia – Janis Misins (Centre for Disease Prevention and Control of Latvia, Rīga); Lithuania – Jelena Isakova (Health Information Centre, Vilnius); Luxembourg – Yolande Wagener (Ministry of Health, Luxembourg); Malta – Miriam Gatt (Department of Health Information and Research, G'Mangia); Netherlands – Jan Nijhuis (Maastricht University Medical Centre, Maastricht); Norway – Kari Klungsøyr (Department of Global Public Health and Primary Care, University of Bergen, Bergen); Poland – Katarzyna Szamotulska (National Research Institute of Mother and Child, Warsaw); Portugal – Henrique Barros (University of Porto, Porto); Romania – Mihai Horga (East European Institute for Reproductive Health, Tirgu Mures); Slovakia – Jan Cap (National Health Information Centre, Bratislava); Slovenia – Natasa Tul (Ljubljana University, Ljubljana); Spain – Francisco Bolúmar (University of Alcala, Madrid); Sweden – Karin Gottvall and Karin Källén (National Board of Health and Welfare, Stockholm); Switzerland – Sylvan Berrut, Mélanie Riggenbach (Swiss Federal Statistical Office, Neuchâtel); UK – Alison Macfarlane (City University London, London). Project coordination: France – Jennifer Zeitlin, Marie Delnord, Mélanie Durox (National Institute of Health and Medical Research [INSERM] U1153, Paris); Netherlands – Ashna Hindori‐Mohangoo (Netherlands Organisation for Applied Scientific Research, Leiden).
