# Supplementary material for: Producing valid statistics when legislation, culture and medical practices differ for births at or before the threshold of survival: report of a European workshop
Source: BJOG. 2019 Nov 6;127(3):314–8. doi: 10.1111/1471-0528.15971 (PMC7003918; doi:10.1111/1471-0528.15971)
Supplement: Supplementary file 1 — Appendix S1 . List of participants in the workshop on ‘Factors affecting the comparability of data sources: Birth & death registration at the limits of viability’ [file BJO-127-314-s001.docx]

# Appendix 1

# List of participants in the workshop on “Factors affecting the comparability of data sources: Birth & death registration at the limits of viability”

# Euro-Peristat Meeting 2018 Kerkrade, Netherlands

## Euro-Peristat country representatives

*Scientific Commitee Members, **Executive Board

**Belgium**

Sophie Alexander*,**

Université Libre de Bruxelles, School of Public Health, Reproductive Health Unit

[salexand@ulb.ac.be](javascript:void(location.href='mailto:'+String.fromCharCode(115,97,108,101,120,97,110,100,64,117,108,98,46,97,99,46,98,101)+'?subject=EURO-PERISTAT%20project')) 

**Bulgaria**

Rumyana Kolarova*
Directorate “National health data and e-health” at the National Center for Public Health and Analysis

r.kolarova@ncpha.government.bg

**Cyprus**

Theopisti Kyprianou

Ministry of Health, Health Monitoring Unit

[ppavlou@moh.gov.cy](javascript:void(location.href='mailto:'+String.fromCharCode(112,112,97,118,108,111,117,64,109,111,104,46,103,111,118,46,99,121)+'?subject=EURO-PERISTAT%20project'))

**Czech Republic**

Petr Velebil*
Institute for the Care of Mother and Child
[VelebilP@seznam.cz](mailto:VelebilP@seznam.cz)

**Denmark**

Laust Mortensen*

Statistics Denmark &

Department of Public Health, University of Copenhagen

[lamo@sund.ku.dk](mailto:lamo@sund.ku.dk)

**Estonia**

Luule Sakkeus*

Estonian Institute for Population Studies, Tallinn University

luule.sakkeus@tlu.ee

**Finland**

**Mika Gissler*,****
THL National Institute for Health and Welfare

[mika.gissler@thl.fi](javascript:void(location.href='mailto:'+String.fromCharCode(109,105,107,97,46,103,105,115,115,108,101,114,64,116,104,108,46,102,105)+'?subject=EURO-PERISTAT%20project'))

**France**

Béatrice Blondel*,**
Inserm UMR 1153, Obstetrical, Perinatal and Pediatric Epidemiology Research Team (Epopé), Center for Epidemiology and Statistics Sorbonne Paris Cité, DHU Risks in pregnancy, Paris Descartes University, Paris, France

[beatrice.blondel@inserm.fr](javascript:void(location.href='mailto:'+String.fromCharCode(98,101,97,116,114,105,99,101,46,98,108,111,110,100,101,108,64,105,110,115,101,114,109,46,102,114)+'?subject=EURO-PERISTAT%20project'))

Marie Delnord**

Inserm UMR 1153, Obstetrical, Perinatal and Pediatric Epidemiology Research Team (Epopé), Center for Epidemiology and Statistics Sorbonne Paris Cité, DHU Risks in pregnancy, Paris Descartes University, Paris, France

[Marie.delnord@inserm.fr](mailto:Marie.delnord@inserm.fr)

Mélanie Durox**

Inserm UMR 1153, Obstetrical, Perinatal and Pediatric Epidemiology Research Team (Epopé), Center for Epidemiology and Statistics Sorbonne Paris Cité, DHU Risks in pregnancy, Paris Descartes University, Paris, France

[Marie.delnord@inserm.fr](mailto:Marie.delnord@inserm.fr)

Jennifer Zeitlin**

Inserm UMR 1153, Obstetrical, Perinatal and Pediatric Epidemiology Research Team (Epopé), Center for Epidemiology and Statistics Sorbonne Paris Cité, DHU Risks in pregnancy, Paris Descartes University, Paris, France

[jennifer.zeitlin@inserm.fr](mailto:jennifer.zeitlin@inserm.fr)

**Germany**

Guenther Heller

Institute for quality assurance and transparency in healthcare (*IQTIG*)

[guenther.heller@iqtig.org](mailto:guenther.heller@iqtig.org)

**Iceland**

Helga Sol Olafsdottir*

Landspitali University Hospital

[helgasol@landspitali.is](mailto:helgasol@landspitali.is)

**Italy**

Maria Lacchei

Pediatric Hospital of Baby Jesus, Unit of Epidemiology

maria.lacchei@opbg.net

Marzia Loghi

National Institute of Statistics

[loghi@istat.it](mailto:loghi@istat.it)

**Latvia**

Irisa Zile*
Centre for Disease Prevention and Control

[Irisa.Zile@spkc.gov.lv](mailto:Irisa.Zile@spkc.gov.lv)

**Lithuania**

Jelena Isakova*
Health Statistics Department, Health Information Centre, Institute of Hygiene

[jelena.isakova@hi.lt](mailto:jelena.isakova@hi.lt)

**Luxembourg**

Esther Arendt

Ministry of Health, Department of Health, Division of Preventive and Social Medicine

[Esther.Arendt@ms.etat.lu](mailto:Esther.Arendt@ms.etat.lu)

Audrey Billy

Ministry of Health, Department of Health, Division of Preventive and Social Medicine

Luxembourg Institute of Health, Department of Population Health

Audrey.Billy@lih.lu

**Malta**

Miriam Gatt*

Dept. of Health Information and Research, National Obstetric Information Systems (NOIS) Register
[miriam.gatt@gov.mt](mailto:miriam.gatt@gov.mt)

**The Netherlands**

Peter Achterberg

National Institute for Public Health and the Environment (*RIVM*)

peter.achterberg@rivm.nl

Jan Nijhuis*
Maastricht University Medical Centre, Department Obstetrics & Gynecology

[jnij@sgyn.azm.nl](mailto:jnij@sgyn.azm.nl)

Chantal Hukkelhoven

Perined

chukkelhoven@perined.nl

**Norway**

Kari Klungsoyr*

Medical Birth Registry of Norway, Norwegian Institute of Public Health
Department of Global Public Health and Primary Care, University of Bergen, Norway

[Kari.Klungsoyr@fhi.no](mailto:Kari.Klungsoyr@fhi.no)

**Poland**

Katarzyna Szamotulska*,**
National Research Institute of Mother and Child, Department of Epidemiology

Ewa Mierzejewska

National Research Institute of Mother and Child, Department of Epidemiology

[ewa.mierzejewska@imid.med.pl](mailto:ewa.mierzejewska@imid.med.pl)

**Portugal**

Henrique Barros*,**
University of Porto Medical School, Department of Hygiene and Epidemiology

[hbarros@med.up.pt](mailto:hbarros@med.up.pt)

**Romania**

Vlad Tica*

East European Institute for Reproductive Health, Faculty of Medicine, University "Ovidius" Constanţa

[vtica@eeirh.org](mailto:vtica@eeirh.org)

**Slovenia**

Nataša Tùl Mandic*

University Medical Centre
Department of Obstetrics&Gynecology - Perinatology Unit

[natasa.tul@guest.arnes.si](mailto:natasa.tul@guest.arnes.si)

Ivan Verdenik

University Medical Centre
Department of Obstetrics&Gynecology - Perinatology Unit

[ivan.verdenik@guest.arnes.si](mailto:ivan.verdenik@guest.arnes.si)

**Spain**

Francisco Bolúmar*
University of Alcalá, Department of Health Sciences and Social Medicine

[francisco.bolumar@uah.es](javascript:void(location.href='mailto:'+String.fromCharCode(102,114,97,110,99,105,115,99,111,46,98,111,108,117,109,97,114,64,117,97,104,46,101,115,32)+'?subject=EURO-PERISTAT%20project'))

Oscar Zurriaga
Directorate of Public Health. Generalitat Valenciana

zurriaga_osc@gva.es

**Sweden**

Karin Källén
The National Board of Health and Welfare
Department of Statistics, Monitoring and Evaluation
Epidemiology Unit
[karin.kallen@socialstyrelsen.se](mailto:karin.kallen@socialstyrelsen.se)

**Switzerland**

Mélanie Riggenbach*

Federal Department of Home Affairs FDHA

Swiss Federal Statistical Office FSO - Section Population Health
[Melanie.Riggenbach@bfs.admin.ch](mailto:Melanie.Riggenbach@bfs.admin.ch)

**United Kingdom**

Alison Macfarlane*,**

City University London, Department of Midwifery and Child Health

[A.J.Macfarlane@city.ac.uk](mailto:A.J.Macfarlane@city.ac.uk )

Lucy Smith
[lucy.smith@leicester.ac.uk](mailto:lucy.smith@leicester.ac.uk)
University of Leicester, MBRRACE-UK collaboration

Rachel Wood

NHS Scotland

[rachaelwood@nhs.net](mailto:rachaelwood@nhs.net)
