# Supplementary material for: Producing valid statistics when legislation, culture and medical practices differ for births at or before the threshold of survival: report of a European workshop
Source: BJOG. 2019 Nov 6;127(3):314–8. doi: 10.1111/1471-0528.15971 (PMC7003918; doi:10.1111/1471-0528.15971)
Supplement: Supplementary file 2 — Box S1 . Clinical scenarios used for discussion of birth and death reporting practices. [file BJO-127-314-s002.pdf]

Box S1. Clinical scenarios used for discussion of birth and death reporting practices.

1. Birth following antepartum fetal death

1.a. Detection at 21^{+5} weeks

A pregnant woman attends an anomaly scan at 21^{+5} weeks gestation. It is a singleton pregnancy. The baby's heartbeat cannot be found during the ultrasound scan and the baby is confirmed to have died. It is not known when the death occurred but a heartbeat was recorded at an appointment at 20^{+2} weeks. The woman is given the option of having the birth induced on that day (21^{+5} weeks); two days later (22^{+0} weeks); or allow the birth to happen naturally.

1.b. Detection at 23^{+5} weeks

A pregnant woman attends a midwifery unit for a routine check at 23^{+5} weeks gestation. It is a singleton pregnancy. The baby's heartbeat cannot be found using a Doppler. An ultrasound scan confirms the baby has died. It is not known when the death occurred but a heartbeat was recorded at an appointment at 22^{+2} weeks. The woman is given the option of having the birth induced on that day (23^{+5}); two days later (24^{+0}); or allow the birth to happen naturally.

1.c. Detection at 23^{+5} weeks within a multiple birth

A pregnant woman expecting twins attends an anomaly scan at 21^{+5} weeks gestation. Twin 1's heartbeat cannot be found during the ultrasound scan and the baby is confirmed to have died. It is not known when the baby died but the last confirmed heartbeat was at the dating scan at 13^{+0}. Twin 2 is alive.

Areas for discussion

- Where would the birth take place – at what gestation do births occur on a gynaecology ward?
- What gestational age is used for registration purposes? Gestational age at birth; Gestational age when death confirmed; other. Does this differ for multiple births?
- How will these factors impact on the parents regarding parental leave and benefits?
- Is registration of births outside statutory registration criteria possible i.e. Optional or informal registration of fetal deaths.
- What international differences may affect data comparability?

2. Birth following extremely preterm rupture of membranes

A pregnant woman arrives at the maternity unit following rupture of membranes and pain at 23^{+5}. She is admitted to the unit. The next day the obstetrician explains that extremely preterm birth is likely. The parents decide that they would rather opt for a termination of pregnancy than risk their child having poor quality of life following extremely preterm birth. The obstetrician agrees and a plan for induction of labour is made. The obstetrician arrives to induce the labour but on examination the woman is already fully dilated and she gives birth at 23^{+6}. The baby shows faint signs of life.

Areas for discussion

- What signs of life define a live birth registration and does the gestation impact on how clinical signs of life are interpreted?
- In what circumstances would resuscitation and active treatment be initiated? Based on gestation at birth? Including/irrespective of parents wishes?
- How is the birth registered if it is a) live birth or b) intrapartum stillbirth?
- If the birth follows termination of pregnancy is the death registered as a stillbirth or neonatal death?
- How will these factors impact on the parents regarding parental leave and benefits?
- Is registration of births outside the criteria possible i.e. Optional or informal registration of births
- What international differences may affect data comparability?
